# Item analysis of the Eating Assessment Tool (EAT-10) by the Rasch model: a secondary analysis of cross-sectional survey data obtained among community-dwelling elders

**DOI:** 10.1186/s12955-020-01384-2

**Published:** 2020-05-13

**Authors:** Tina Hansen, Annette Kjaersgaard

**Affiliations:** 1 Division of Occupational Therapy, Faculty of Health and Technology, Copenhagen University College, Sigurdsgade 26, 2200 N Copenhagen, Denmark; 2 Department for Education, Hammel Neurorehabilitation Centre and University Research Clinic, Voldbyvej 15, 8450 Hammel, Denmark

**Keywords:** Deglutition disorders, Psychometrics, Patient reported outcome measures, Reliability, Validity, Screening

## Abstract

**Background:**

The Eating Assessment Tool (EAT-10) is increasingly used to screen for self-perceived oropharyngeal dysphagia (OD) in community-dwelling elders. A summated EAT-10 total score ranges from 0 to 40, with a score ≥ 3 indicative of OD. When using cut-points of a summated score, important requirements for the measurements are specific objectivity, validity, and reliability. Analysis by the Rasch model allows investigation of whether scales like EAT-10 satisfy these requirements. Currently, a few studies have found that EAT-10 responses from clinical populations with OD do not adequately fit the Rasch model.

**Purpose:**

The aim of this study was to determine whether measurements by EAT-10 fit the Rasch model when applied in screening self-perceived OD in non-clinical populations.

**Methods:**

Secondary analysis was conducted on data from a cross-sectional survey of community-dwelling elders living in a municipal district of Tokyo, Japan, in which 1875 respondents completed the Japanese version of EAT-10 (J-EAT-10). Data were cleaned and recoded for the purpose of the analysis in this study, which resulted in inclusion of J-EAT-10 responses from 1144 respondents. Data were analyzed using RUMM2030 and included overall model fit, reliability, unidimensionality, threshold ordering, individual item and person fits, differential item functioning, local item dependency, and targeting.

**Results:**

The analysis identified that the response categories from zero to four were not used as intended and did not display monotonicity, which necessitated reducing the five categories to three. Considerable floor effect was demonstrated and there was an inappropriate match between items’ and respondents’ estimates. The person separation reliability (PSI = 0.65) was inadequate, indicating that it is not possible to differentiate between different levels of OD. Several items displayed misfit with the Rasch model, and there were local item dependency and several redundant items.

**Conclusions:**

J-EAT-10 performed less than optimally and exhibited substantial floor effect, low reliability, a rating scale not working as intended, and several redundant items. Different improvement strategies failed to resolve the identified problems. Use of J-EAT-10 in population-based surveys cannot therefore be recommended. For such purpose, alternative screening tools of self-perceived OD should be chosen or a new one should be developed and validated.

## Background

Oropharyngeal dysphagia (OD), which impairs swallowing efficiency and safety, is common in old age as a result of several underlying processes and diseases [1–4]. OD increases the risk of malnutrition and dehydration [5], aspiration pneumonia [6], depression and anxiety [7], and decreased quality of life [8], as well as increasing health care expenditure and utilization [9, 10]. It is recognized that community-dwelling elders are at risk of developing OD, with an estimated mean prevalence of 15% across high quality studies included in a recent meta-analysis [4]. With an aging population, OD is an important and serious current and future health issue necessitating identification of elders at risk [1, 9, 10]. To take a proactive approach for avoiding the health-related and economic consequences of OD, systematic screening among community-dwelling elders is recommended [1–3, 10].

The Eating Assessment Tool (EAT-10) [11], a patient reported outcome measure (PROM) of self-perceived symptoms of OD, is recommended as an easy to use and quick screening tool for OD [2, 3]. EAT-10 was developed and validated for use in estimating initial OD severity and changes in response to therapy [11]. Supplemental file 1 shows the content of EAT-10, which comprises ten items to be rated on a 5-point response scale (0–4) with labels at the extremes of ‘0 = No problem’ and ‘4 = Severe problem’, resulting in a range of 0–40 [11]. EAT-10 has been translated into several different language versions published by the Nestle Nutrition Institute [12], and it is increasingly used as a screening tool for OD in clinical populations [3, 13–18] as well as in non-clinical populations of community-dwelling elders [19–23]. The diagnostic efficiency [24] of EAT-10 in terms of sensitivity (e.g., identifying persons with OD) and specificity (e.g., identifying persons without OD) has been quantified for different cut-off points. For example, it is suggested that an EAT-10 total score ≥ 2 [25] or ≥ 3 is indicative of OD [11], and that a total score > 15 is indicative of aspiration risk [26]. When quantifying the diagnostic efficiency of a scale, the summated score must accurately reflect what is being measured [24]. In the case of EAT-10, we obtain a measure of self-perceived OD severity, which is not directly observed and is therefore regarded as a latent variable. This is the opposite of a manifest variable, which can be directly measured or observed [27], such as videofluoroscopy swallowing evaluation [28]. Using a summated score of EAT-10 responses, it is therefore necessary to determine whether the items contribute to one single dimension of lower or higher OD severity. Hence, an important requirement of EAT-10 is that it should have specific objectivity [29], which implies invariance - the comparison between any two persons is independent of the rating scale items used and vice versa [27, 29, 30].

Within modern item response theory, the Rasch model has been considered the gold standard against which scales summarizing item responses can be tested [27, 29, 30]. Analysis by the Rasch model is a statistical method that allows detailed information on the performance of a set of item responses as a measure of a latent variable [27]. The Rasch model expresses the association between observed (actual) item performance and underlying ability (unobserved) or a latent variable (i.e., OD severity in the case of EAT-10). Hence, the set of items in EAT-10 must satisfy certain requirements to fit the Rasch model before it can be considered to measure a continuous latent variable of less or more [27, 29, 30], namely:

- Unidimensionality: the items of a scale should measure only one latent variable (i.e., all EAT-10 items measure aspects of OD severity).
- Monotonicity: the scale items function hierarchically from easy to difficult, and the probability of a high item score should increase with increasing values of the latent variable (i.e., the probability of giving a score that reflects a swallowing problem increases with high EAT-10 total scores).
- Homogeneity: The rank order of the items from easy to difficult should be the same for all respondents, regardless of their level for the latent variable (i.e., the order of EAT-10 items according to the severity of the problem they express is the same for all respondents, regardless of their level on the scale, as reflected in the EAT-10 total score; the easiest problem to have is easiest for all respondents and vice versa).
- Local independency: the items of a scale must be conditionally independent given the latent variable (i.e., the rating of any one problem should depend only on the level of the scale, as reflected in the EAT-10 total score and not the rating of any other items).
- Absence of differential item function (DIF): the items should be conditionally independent of exogenous variables given the latent variable (i.e., the EAT-10 items should function equally for subgroups of respondents, for example male and female).

If these requirements are met by the items in EAT-10, the obtained measurement is assumed to be reliable and construct valid [27, 29, 30], and will provide ideal measurement of OD severity. Accordingly, the raw score can be regarded as a sufficient statistic for the estimated person parameter, and measurement by the scale is considered specifically objective [27, 29, 30].

After publication of EAT-10 [11], studies have found that, when used in clinical populations, EAT-10 does not fit the Rasch model sufficiently [16–18] and demonstrates low reliability, with several items not contributing adequately to a latent unidimensional variable [16–18], DIF by OD severity [16, 17], gender and different language versions [16], lack of monotonicity of the response scale [16, 18], and substantial floor effects (i.e., no problems) of 23% [16] and 57% [18]. If EAT-10 is applied in population-based screening among community-dwelling elders, larger floor effects might occur, since OD prevalence is lower in non-clinical compared to clinical populations [4]. It is worth noting that the performance of a screening test such as EAT-10 is dependent on the prevalence of the condition in question [24]. EAT-10 was developed and validated to document initial OD severity and monitor response to treatment in symptomatic patients [11]; it was not designed for population-based screening in the wider community. With the increased use of EAT-10 in the wider community [19–23], analysis by the Rasch model of EAT-10 responses obtained from non-clinical populations is needed. The aim of this study was therefore to evaluate whether measurements by EAT-10 are reliable, valid, and upholds specific objectivity when applied as a screening tool for detecting OD among community-dwelling elders.

## Method

Analysis by the Rasch model was performed as a secondary data analysis of an existing dataset available as an information file (Excel format) supporting a cross-sectional survey on OD prevalence among elders living in a municipal district of Tokyo, Japan, by Igarashi et al. [22]. This is an open access article distributed under the terms of the Creative Commons Attribution License, which permits unrestricted use, distribution, and reproduction in any medium, provided the original author and source are credited. The survey by Igarashi et al. included the Japanese version of EAT-10 (J-EAT-10) [31], was conducted with formal approval and is described in detail in Igarashi et al. [22]. Since the current study involves a secondary analysis of freely available data, formal ethical approval was not needed.

### Source of data and data cleaning

Supplemental file 2 presents the codes for the original and current datasets. For the purpose of the analysis by the Rasch model, the dataset included: gender, age in years stratified by quartiles, functional level stratified into independent and dependent respondents, and item responses on J-EAT-10. In total, the original Excel file included data from 1875 anonymized respondents [32]. Of these, 731 respondents were removed from the current dataset due to incomplete responses on J-EAT-10 (*N* = 378) or assignment of values without codes for the variable “functional level” (*N* = 353). Accordingly, 1144 responses were included for the analysis by the Rasch model.

### Analysis by the Rasch model

The analysis by the Rasch model was performed using the RUMM2030 software [33], which integrates a conditional pairwise maximum likelihood algorithm for the parameter estimations [30, 34]. In the case of J-EAT-10, the Rasch model specifies that the probability of a response of 0, 1, 2, 3 or 4 is a logistic function of the difference between the respondent’s level for the measured variable (i.e., severity of OD) and the level represented by the item. Logits (log-odd units) are the unit of measurement for reporting the relative differences between the estimates of a person’s level and item difficulties, and are an equal interval level of measurement. Persons (i.e., respondents) and items are located on the same measurement scale, with the mean item location set at zero logits. Accordingly, the ordinal scores from the J-EAT-10 items are expressed as linear measures, where negative values reflect easy items and a lower degree of OD severity and positive values reflect difficult items and a higher degree of OD severity [27, 29, 30].

The analysis followed recommended procedures [27, 35–37] and was carried out on responses from the full sample as well as separately for the independent and dependent respondents. Model fit was examined statistically and graphically, and carried out for items, persons, and different ability groups (i.e., class intervals) according to their locations on the measured variable. Ideally, the class intervals should be approximately equally distributed with at least 50 persons in each [34].

### Person and item level fit to the Rasch model

Statistically, model fit was examined using standardized fit residual values, which express the differences between observed responses to the J-EAT-10 items and those expected by the model, and by analyzing them by means of chi-squared (χ^2^) statistics and analysis of variance (ANOVA) of the residuals across class intervals. Fit residuals values between ±2.5 for persons and items indicates model fit [27, 30, 34, 36, 37]. High item fit residuals signify under discrimination and might reflect multidimensionality, while low fit residuals signify over discrimination and might reflect potential redundancy or item dependency within the item set [27, 30]. Chi-squared statistics and ANOVA should reflect non-significant (Bonferroni adjusted) deviations from model expectations [27, 30, 34]. Item fit was also examined via visual inspections using graphs of observed item responses for each class interval plotted against the model expectations, which are displayed as an item characteristic curve (ICC) [27, 30, 34].

Local independence was investigated using a residual correlation matrix of the items. Local item dependence (LID) was evident by item residual correlations above 0.2 of the average correlation, reflecting that the entire correlation between the items is not captured by the latent variable [38]. This might happen when the content of a previous item affects responses to a subsequent (dependent) item [27, 30].

Differential item functioning (DIF) refers to item bias that occurs when subgroups with a similar level for the measured variable have a different response pattern to an item [30, 35]. DIF was examined by gender, age, and functional level. For the analysis, a two-way ANOVA on the residuals for each item across the subgroups and across the class intervals is applied. DIF can occur as uniform DIF, where item responses differ uniformly across the measured variable (i.e., a main effect) or as non-uniform DIF, where differences in item responses between subgroups vary across the measured variable (i.e., an interaction effect). The Bonferroni correction was used to adjust for multiple testing, keeping the type I error to 5% [34].

### The scoring structures

J-EAT-10 consists of polytomous items with five response categories ordered to reflect an increasing amount of OD [11]. The boundaries between adjacent categories are called thresholds. As the number of thresholds is one less than the number of response categories, there are four thresholds for each item, which reflect positions on the latent variable where either of the adjacent responses is equally probable [27, 30, 34]. For fit to the Rasch model, monotonicity by means of ordered thresholds is expected, which implies that the transition from one score to the next is consistent with the increase in the latent variable [27]. Monotonicity was examined using the item thresholds parameters, a threshold map, and category probability curves. In addition, further analysis was performed by examining the category response frequencies. Before performing analysis by the Rasch model of polytomous data, a choice between two different parameterization methods is undertaken [27, 34], namely the Rating Scale Model (RSM) [39] or the Partial Credit Model (PCM) [40]. In the RSM, only one set of thresholds across all items is estimated, while in the PCM thresholds for each of the items are estimated [27]. Accordingly, the PCM contains a lot more information and is a more complex model because additional parameters are estimated compared to the RSM [27]. In RUMM2030, Fisher’s likelihood ratio test is available to assess the efficiency of the two different parameterizations. If the test is significant, it indicates that the PCM should be adopted [27, 34].

### Overall fit to the Rasch model

Overall model fit is provided in RUMM2030 by summary fit residual statistics for items and persons, which should approach a standardized mean value of zero and an SD of 1.0, and by a summary item χ^2^ statistic, which should be non-significant (*p* > 0.05) reflecting homogeneity of the items across the different class intervals [27, 30, 34]. In addition, reliability and unidimensionality of the scale are reported.

Reliability was examined using Cronbach’s alpha (α) and the Person Separation Index (PSI), the Rasch equivalent of Cronbach’s α, except that it is calculated from the logit scale person estimates [27, 30, 34]. It is suggested that α/PSI ≥ 0.90 = excellent, 0.90 > α/PSI ≥ 0.80 = good, 0.8 > α/PSI ≥ 0.7 = acceptable, 0.7 > α/PSI ≥ 0.6 = questionable, 0.6 > α/PSI ≥ 0.5 = poor, and α/PSI < 0.5 = unacceptable [41, 42]. The PSI indicates the power of the latent variable to discriminate among persons and reflects the power of the fit statistics, which RUMM2030 displays as excellent, good, reasonable, low, or too low. If the PSI is not acceptable, the top measure cannot be statistically distinguished from the bottom measure with any confidence and the obtained fit statistics may not be reliable because of too large an error variance [27, 34].

Unidimensionality is defined as the absence of any meaningful pattern in the residuals, which was assessed by Principal Component Analysis [27, 30, 34]. Based on the loading between items and the first residual factor, two subsets of items consisting of items with positive and negative loadings were identified. The differences in location estimates for each person from these two subsets of items were investigated using a series of t-tests. Unidimensionality was confirmed if less than 5% of the sample showed a significant difference in location estimates [27, 30, 34].

### Targeting

Targeting is defined as the extent to which the range of the measure matches the range of the measure in the study sample. To be considered a well-targeted rating scale, J-EAT-10 should have item and person mean locations of around zero and have enough items of varied degrees of OD, matching the spread of scores among respondents [27, 30, 34]. Targeting was examined using a person-item thresholds distribution map, which visually depicts person locations against item-threshold locations [34]. If J-EAT-10 is poorly targeted, respondents may report having no problems (floor effect) or severe problems (ceiling effect) [27].

### Improvement strategies

RUMM2030 provides opportunities to apply improvement strategies to achieve fit to the Rasch model [34]. Before deciding which strategies to employ, the overall model fit statistics, the item level fit statistics, and visual inspections of the ICCs as well as the category response frequencies and threshold ordering were taken together. Disordered thresholds may be resolved by combining adjacent categories [27, 34], mis fitting items or persons can be removed [27, 30, 34], and uniform DIF can be addressed by splitting the item into group specific items. Non-uniform DIF is usually removed, as it reflects misfit to the model [30, 34, 35]. LID can be addressed by grouping local dependent items into a “super-item” to absorb the impact of LID [27, 30].

### Sample size

For a well targeted rating scale, a sample size of around 250–500 usually provides accurate and stable person and item estimates as well as a good balance for statistical interpretation of the fit statistics [43, 44]. Since the current dataset comprises a sample size of 1144, there is a risk of type I error associated with the fit statistics and a post-hoc downward sample size adjustment might be needed [44]. However, the reported floor effect, and thus the high percentages of respondents with a minimum EAT-10 total score of 0 from clinical populations [16, 18], ought to be considered. In Rasch modeling, such total scores are regarded as extreme person scores, which contain no information for rank ordering of persons and items or for estimating the threshold parameters. In RUMM2030, extreme persons are by default omitted from the estimation of the item location and the test-of-fit statistics due to lack of precision involved with the parameter estimates [27, 34]. Thus, the effective sample size for Rasch modeling will always be smaller than the original sample size [27]. Since current analysis included responses obtained in a non-clinical population, the presence of extreme person scores was expected, and the magnitude was assessed before deciding whether it was necessary to adjust the sample size.

## Results

### Verification of model and sample size

The likelihood ratio test was significant (χ^2^ (df) = 317.26 (26), *p* < 0.001), indicating that the PCM should be adopted. The initial analysis of the full sample (*N* = 1144) found 483 respondents with an EAT-10 total score of 0 resulting in 42% extreme scores. Hence, an effective sample size of 661 respondents was included without downward adjustment.

### Overall fit to the Rasch model

Table 1 shows the overall fit statistics. The initial analysis of the full sample (Table 1, analysis 1) showed significant item-trait interaction (χ^2^ (df) = 485.48 (40), *p* < 0.001) and a fit residual mean value (SD) for items of − 0.66 (4.16), both indicating misfit of the responses to the Rasch model. The fit residual mean (SD) for persons was − 0.30 (1.08), indicating no serious misfit. The t-tests suggested unidimensionality, with only 2.27% statistically significant different person estimates based on the two most divergent subsets of items within the J-EAT-10 scale. The PSI without extreme scores was 0.65, the power of analysis of fit was good, and Cronbach’s α was 0.85 indicating good reliability. As shown in Table 1, the overall fit statistics persisted to indicate model misfit when separately analyzing the data from the independent respondents (analysis 2) and the dependent respondents (analysis 3). Extreme scores were present for 53% of the independent respondents and 30% of the dependent respondents.

**Table 1:** Analysis by the Rasch model - overall fit statistics for J-EAT-10

Scale analysis	% Extr.	Fit residuals mean (SD)		Item-trait interaction		Reliability		Unidi-mensionality
		Item	Person	*χ*^2^ (df)	*P*	PSI / α	Power of fit	T-test %
**Initial analyses**								
1. Full sample (*N* = 1144)	42%	−0.66 (4.16)	− 0.30 (1.08)	485.48 (40)	< 0.001	0.65 / 0.85	Good	2.27%
2. Independent group (*N* = 594)	53%	− 0.82 (2.13)	−0.37 (0.86)	175.92 (30)	< 0.001	0.32 / 0.72	Too low	0.72%
3. Dependent group (*N* = 550)	30%	−0.19 (3.53)	−0.25 (1.17)	293.28 (40)	< 0.001	0.74 / 0.87	Good	1.83%
**Analysis after reducing to three scoring categories (full sample,*N*** **= 1144)**								
4. Scoring structure: 00122^a^	65%	−0.89 (3.21)	−0.25 (0.77)	284.01 (40)	< 0.001	0.38 / 0.76	Low	1.73%
5. Scoring structure: 01112	42%	−1.73 (4.72)	−0.35 (0.90)	416.01 (40)	< 0.001	0.69 / 0.83	Good	1.21%
6. Scoring structure: 01122	42%	−1.90 (4.34)	−0.38 (1.01)	448.90 (40)	< 0.001	0.66 / 0.83	Good	2.27%
7. Scoring structure: 01222	43%	−0.78 (4.26)	−0.26 (1.11)	439.37 (40)	< 0.001	0.65 / 0.89	Reasonable	0.76%
8. Scoring structure: 00112	65%	−0.56 (4.04)	−0.24 (0.74)	225.24 (30)	< 0.001	0.49 / 0.76	Low	1.98%
***Optimal values < 15%***		***0 (< 1.00)***	***0 (< 1.00)***		***> 0.05***	***> 0.70***	***< 5%***	

*J-EAT-10* Japanese version of the Eating Assessment Tool, *% Extr.* percentage of extreme scores, *χ*^*2*^ Chi-square, *SD* standard deviation, *df* degrees of freedom, *PSI* Person Separation Index, *α* Cronbach’s alpha, *CI* confidence interval

^a^Scoring structure suggested by Cordier et al. [16]

### Item level fit to the Rasch model

**Fig. 1:**
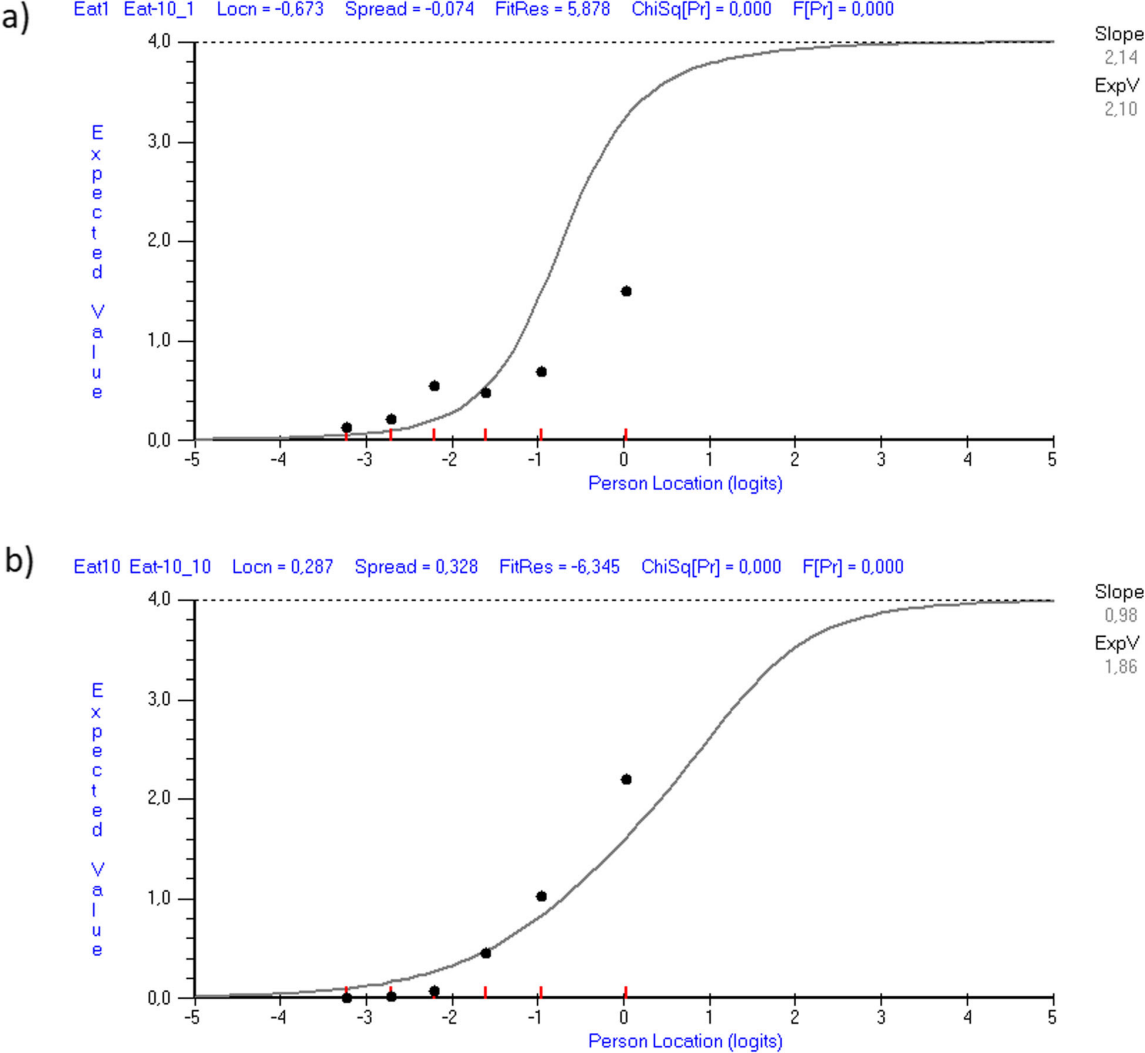
Item characteristic curves (ICC) of two mis fitting items of J-EAT-10. ICC plot for two items. Based on the sample size, persons (respondents) are divided into six ability groups (class intervals with at least 50 persons in each). The curved line represents the expected scores for the item, and the dots represent the observed scores for the class intervals at the different levels of the measured variable (*self-perceived OD severity*). **a** The ICC plot for item 1 with a high positive and significant fit residual of 5.9. The observed scores form a flatter curve than the expected scores, which indicates that this item is under discriminating and might reflect multidimensionality. **b** The ICC plot for item 10 with a high negative and significant fit residual of − 6.3. The observed scores form a steeper curve than the expected scores, which indicates that this item is over discriminating and might reflect potential redundancy or dependency within the item set

Table 2 shows the fit statistics at item level. The analysis of the full sample showed that items 1 and 9 displayed significant positive fit residuals > 2.5, which indicates multidimensionality, as illustrated in Fig. 1a) for item 1. Items 3, 4, 6 and 10 showed significant negative fit residuals, indicating redundancy or dependency within the item set. This is illustrated in Fig. 1b) for item 10. As shown in Table 3, no items displayed uniform or non-uniform DIF by gender, age, or functional level. LID was found for item pair 5 and 6 (residual correlation: *r* = 0.29). When analyzing the independent and dependent respondents separately, the item level fit statistics approached the findings for the full sample (Table 2).

**Table 2:** Individual item location and fit statistics for J-EAT-10

Item-abbreviated	Loc	SE	FR	*χ*^2^ (df)	*P*	F (df)	*P*
**Full sample**							
1. Lose weight	−0.67	0.05	**5.9**	186.01 (5)	**< 0.001**	20.52 (5.655)	**< 0.001**
2. Go out meals	1.00	0.07	0.9	5.93 (5)	0.313	0.92 (5655)	0.463
3. Liquids effort	0.04	0.06	**−3.0**	19.33 (5)	**0.002**	4.64 (5655)	**< 0.001**
4. Solids effort	−0.29	0.06	**−3.5**	37.34 (5)	**< 0.001**	8.96 (5655)	**< 0.001**
5. Pills effort	−0.51	0.05	−0.1	9.35 (5)	0.096	1.45 (5655)	0.204
6. Painful	0.10	0.06	**−5.7**	62.73 (5)	**< 0.001**	25.20 (5655)	**< 0.001**
7. Pleasure eat	−0.36	0.06	1.6	28.77 (5)	**< 0.001**	4.51 (5655)	**< 0.001**
8. Stick throat	−0.11	0.06	−1.5	32.71 (5)	**< 0.001**	7.39 (5655)	**< 0.001**
9. Cough	0.52	0.06	**5.1**	31.30 (5)	**< 0.001**	5.33 (5655)	**< 0.001**
10. Stressful	0.29	0.07	**−6.3**	72.01 (5)	**< 0.001**	33.95 (5655)	**< 0.001**
**Independent respondents**							
1. Lose weight	−0.62	0.07	1.7	41.12 (3)	**< 0.001**	10.88 (3274)	**< 0.001**
2. Go out meals	0.35	0.13	0.8	1.77 (3)	0.622	0.54 (3274)	0.656
3. Liquids effort	0.42	0.12	−2.3	12.37 (3)	0.006	5.22 (3274)	**0.002**
4. Solids effort	0.02	0.09	−2.0	15.31 (3)	**0.002**	5.69 (3274)	**0.001**
5. Pills effort	−0.47	0.08	0.4	4.76 (3)	0.190	1.19 (3274)	0.313
6. Painful	0.06	0.10	**−3.6**	30.93 (3)	**< 0.001**	21.86 (3274)	**< 0.001**
7. Pleasure eat	−0.28	0.09	0.0	11.88 (3)	0.008	2.91 (3274)	0.035
8. Stick throat	0.27	0.10	−1.0	12.80 (3)	**0.005**	4.08 (3274)	0.007
9. Cough	−0.24	0.09	1.9	16.06 (3)	**0.001**	5.29 (3274)	**0.001**
10. Stressful	0.50	0.12	**−4.1**	28.93 (3)	**< 0.001**	20.83 (3274)	**< 0.001**
**Dependent respondents**							
1. Lose weight	−0.61	0.06	**5.9**	122.30 (3)	**< 0.000**	19.71 (3379)	**< 0.001**
2. Go out meals	0.96	0.08	0.9	1.90 (3)	0.594	0.44 (3379)	0.723
3. Liquids effort	0.04	0.07	−1.8	8.49 (3)	0.037	3.14 (3379)	0.025
4. Solids effort	−0.36	0.07	**−2.7**	25.42 (3)	**< 0.000**	11.50 (3379)	**< 0.001**
5. Pills effort	−0.45	0.07	−0.8	8.56 (3)	0.036	3.05 (3379)	0.029
6. Painful	0.14	0.08	**−4.2**	37.25 (3)	**< 0.000**	25.20 (3379)	**< 0.001**
7. Pleasure eat	−0.35	0.07	1.9	16.02 (3)	**0.001**	3.43 (3379)	0.017
8. Stick throat	−0.14	0.07	−1.0	13.25 (3)	**0.004**	5.32 (3379)	**0.001**
9. Cough	0.53	0.07	**4.6**	19.72 (3)	**< 0.000**	5.00 (3379)	**0.002**
10. Stressful	0.24	0.08	**−4.7**	40.32 (3)	**< 0.000**	30.76 (3379)	**< 0.001**
***Optimal value***			**<±2.5**		**≥0.05**^a^		**≥0.05**^a^

Bold indicates misfit or violation of the Rasch model

*J-EAT-10* Japanese version of the Eating Assessment Tool, *Loc.* location, *SE* standard error, *FR* fit residual, *χ*^*2*^ Chi-square, *df* degrees of freedom, *F* F-statistic

^a^Bonferroni adjusted *p* = 0.005 for ten items

**Table 3:** Summary of differential item function (DIF) by gender, age, and functional level for J-EAT-10

Item (abbreviated)	Gender^a^				Age^b^				Functional level^c^			
	Uniform		Non-uniform		Uniform		Non-uniform		Uniform		Non-uniform	
	F(df)	*p*	F(df)	*p*	F(df)	*p*	F(df)	*p*	F(df)	*p*	F(df)	*p*
1. Lose weight	0.35 (1)	0.556	1.87 (5)	0.097	0.06 (3)	0.979	1.22 (15)	0.250	0.63 (3)	0.598	1.05 (15)	0.405
2. Go out meals	0.07 (1)	0.789	1.22 (5)	0.296	3.22 (3)	0.022	1.22 (15)	0.248	3.98 (3)	0.008	0.62 (15)	0.857
3. Liquids effort	0.86 (1)	0.353	1.44 (5)	0.207	0.53 (3)	0.664	0.98 (15)	0.474	2.11 (3)	0.097	0.56 (15)	0.904
4. Solids effort	0.21 (1)	0.645	1.06 (5)	0.381	0.27 (3)	0.844	0.82 (15)	0.661	1.04 (3)	0.373	0.60 (15)	0.879
5. Pills effort	4.24 (1)	0.040	0.03 (5)	1.000	1.21 (3)	0.304	1.46 (15)	0.115	2.05 (3)	0.105	0.83 (15)	0.640
6. Painful	2.48 (1)	0.116	0.95 (5)	0.449	0.63 (3)	0.597	1.46 (15)	0.114	1.35 (3)	0.258	0.67 (15)	0.819
7. Pleasure eat	0.50 (1)	0.481	0.14 (5)	0.984	1.39 (3)	0.245	0.31 (15)	0.995	0.93 (3)	0.426	1.15 (15)	0.307
8. Stick throat	2.85 (1)	0.092	1.35 (5)	0.243	0.19 (3)	0.905	0.83 (15)	0.640	0.57 (3)	0.634	0.70 (15)	0.786
9. Cough	2.26 (1)	0.133	0.65 (5)	0.658	0.43 (3)	0.731	0.85 (15)	0.627	1.19 (3)	0.312	1.33 (15)	0.175
10. Stressful	0.35 (1)	0.555	1.62 (5)	0.151	1.76 (3)	0.153	0.66 (15)	0.828	1.95 (3)	0.121	0.19 (15)	1.000

Significance level *p* < 0.05 (Bonferroni adjusted *p* = 0.002)

*J-EAT-10* Japanese version of the Eating Assessment Tool, *df* degrees of freedom

^a^Dichotomized into male (*N* = 475) / female (*N* = 669)

^b^Stratified by quartiles: ≤ Q1 ~ 60–70 years (*N* = 313) / ≤ Q2 ~ 71–77 years (*N* = 298) / ≤ Q3 ~ 78–83 years (*N* = 2 63) / > Q ~ 384–99 years (*N* = 270)

^c^Stratified into four groups: independent (*N* = 594) / dependent of care 25–89 min per day (*N* = 265) / dependent of care 90–109 min per day (*N* = 147) /dependent of care ≥110 min per day (*N* = 138)

### The scoring structures

**Fig. 2:**
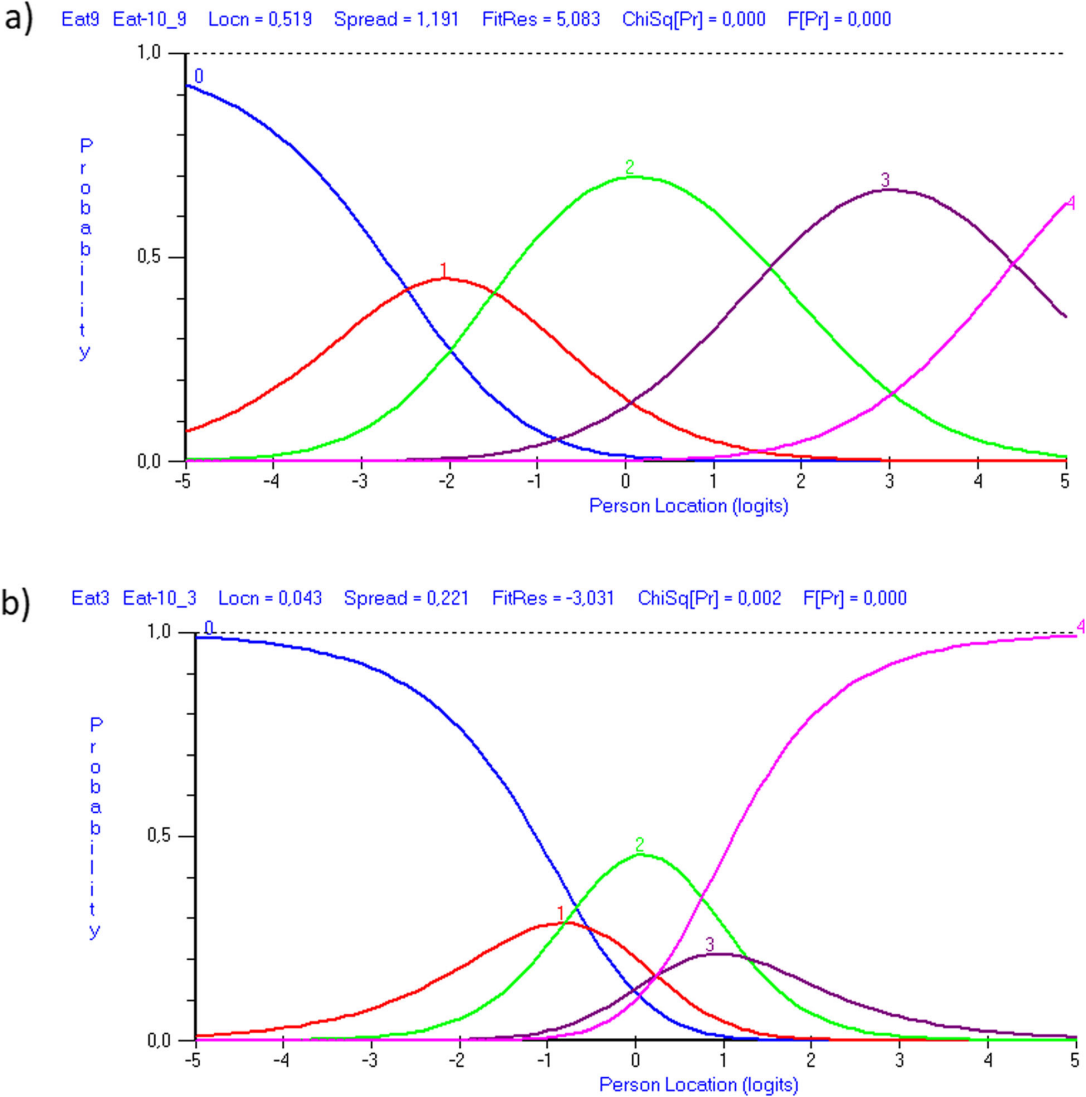
Category probability curves for two items of J-EAT-10. The y-axis represents the probability of observing each category of the five response options on J-EAT-10 at each level of item difficulty on the x-axis. Each colour corresponds to the different response options: 0 = blue, 1 = red, 2 = green, 3 = purple, 4 = pink. The intersections of adjacent curves are the thresholds. **a** The category probability curves for item 9 (Cough) with ordered thresholds. The responses to this item are distributed in a logical progressive order, and as a respondent’s OD severity increases, so the probability of achieving the next score increases. **b** The category probability curves for item 3 (Liquids effort) with disordered thresholds. The responses to this item are not distributed in a logical progressive order, and a score of 1 or 3 is never probable

Table 4 shows that most items obtained scores of 0 or 1, and they displayed disordered thresholds during analysis of the full sample as well as of the independent and dependent respondents. Figure 2 illustrates category probability curves for item 9 with ordered thresholds and for item 3 with disordered thresholds.

**Table 4:** Category frequencies and item threshold parameters for each item of J-EAT-10

	Full sample					Independent group					Dependent group				
**Item (abbreviated)**	**Category frequencies**					**Category frequencies**					**Category frequencies**				
	0	1	2	3	4	0	1	2	3	4	0	1	2	3	4
1. Lose weight	497	72	50	23	19	229	19	19	8	3	268	53	31	15	16
2. Go out meals	527	84	37	11	2	251	21	4	2	0	276	63	33	9	2
3. Liquids effort	486	92	67	8	8	232	28	17	0	1	254	64	50	8	7
4. Solids effort	393	145	100	13	10	195	52	27	4	0	198	93	73	9	10
5. Pills effort	389	122	128	7	15	191	44	37	3	3	198	78	91	4	12
6. Painful	449	114	83	9	6	216	41	18	2	1	233	73	65	7	5
7. Pleasure eat	451	124	60	14	12	218	41	12	5	2	233	83	48	9	10
8. Stick throat	348	181	125	1	6	165	80	33	0	0	183	101	92	1	6
9. Cough	204	256	188	12	1	90	129	57	2	0	114	127	131	10	1
10. Stressful	472	117	61	6	5	221	43	13	1	0	251	74	48	5	5
	**Thresholds**					**Thresholds**					**Thresholds**				
	0/1	1/2		2/3	3/4	0/1	1/2	2/3	3/4		0/1	1/2	2/3		3/4
1. Lose weight	***0.62***	***−0.54***		***−0.06***	***−0.03***	***1.16***	***−0.99***	***−0.22***	***0.05***		***0.42***	***−0.30***	***0.03***		***−0.15***
2. Go out meals	***−1.22***	***−1.31***		***− 0.50***	***3.04***	***0.19***	***0.19***	***−0.96***	***0.58***		−1.42	−1.38	− 0.18		2.97
3. Liquids effort	***−0.58***	***− 0.85***		***1.24***	***0.20***	***− 0.47***	***− 1.01***	***2.30***	***− 0.82***		***− 0.70***	***− 0.85***	***0.99***		***0.56***
4. Solids effort	***− 1.20***	***− 0.58***		***1.33***	***0.45***	***− 1.05***	***− 0.53***	***0.83***	***0.75***		***− 1.24***	***− 0.54***	***1.58***		***0.21***
5. Pills effort	***−0.75***	***− 0.84***		***2.18***	***−0.59***	***− 0.36***	***−0.61***	***1.47***	***−0.49***		***− 0.92***	***−0.90***	***2.55***		***−0.73***
6. Painful	***−1.08***	***−0.83***		***1.32***	***0.59***	***−0.70***	***−0.24***	***1.04***	***−0.10***		***−1.19***	***−1.01***	***1.45***		***0.74***
7. Pleasure eat	***−0.61***	***0.02***		***0.71***	***−0.12***	***−0.26***	***0.48***	***−0.03***	***− 0.18***		***−0.73***	***− 0.09***	***1.02***		***− 0.21***
8. Stick throat	***−1.71***	***−0.90***		***3.99***	***−1.38***	***− 1.90***	***− 0.72***	***2.56***	***0.06***		***− 1.59***	***−1.00***	***3.82***		***−1.23***
9. Cough	−3.00	−2.02		1.12	3.90	***−2.18***	***−0.86***	***2.33***	***0.71***		−2.79	−2.11	1.07		3.84
10. Stressful	***−1.16***	***−0.63***		***1.48***	***0.32***	***−1.08***	***−0.28***	***1.15***	***0.20***		***−1.12***	***−0.71***	***1.59***		***0.24***

Bold and italics indicate disordered thresholds

*J-EAT-10* Japanese version of the Eating Assessment Tool

### Targeting

**Fig. 3:**
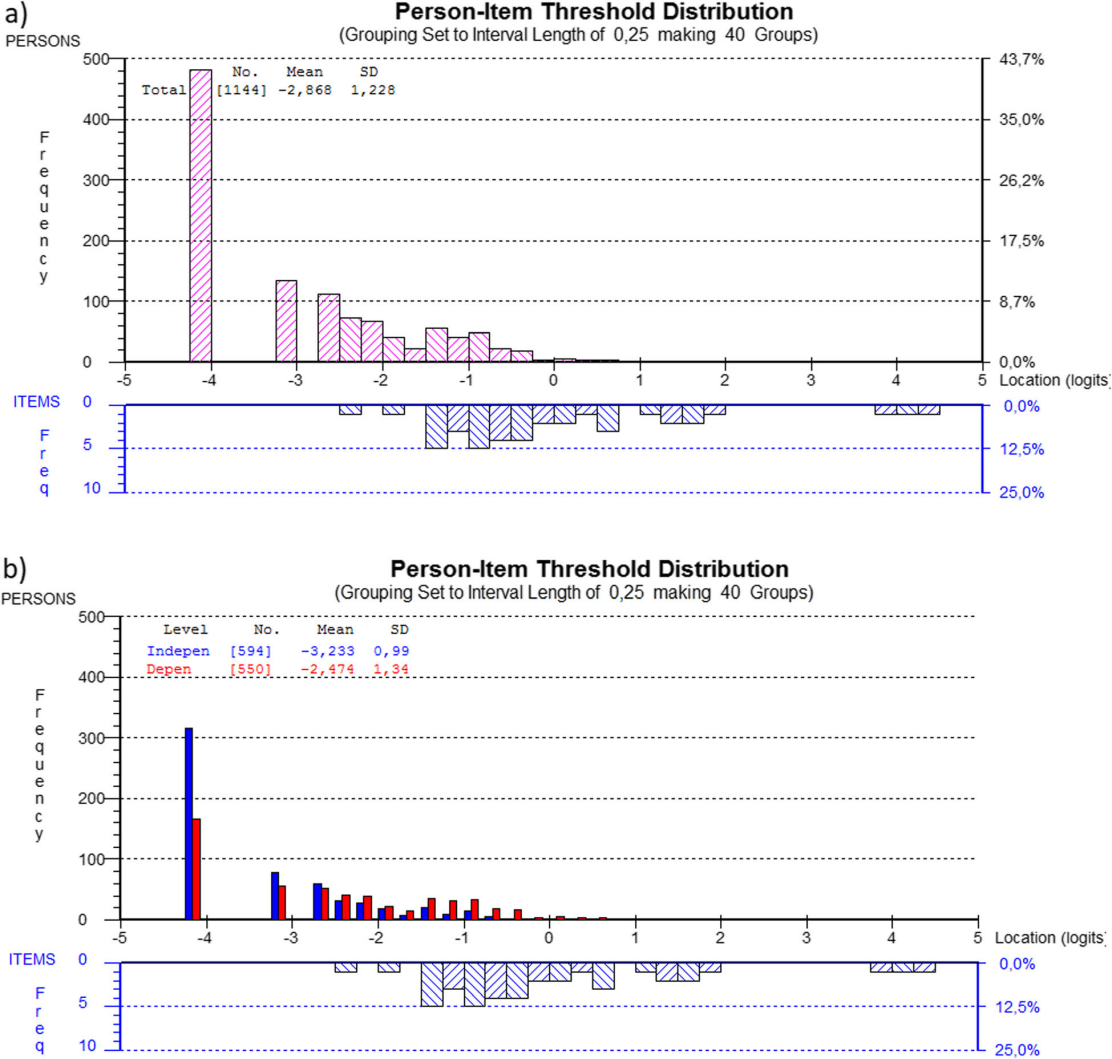
Person-item threshold distribution of the J-EAT-10 responses. The x-axes display location of item thresholds (lower half) and location of respondents’ summated OD severity on J-EAT-10 (upper half). The y-axes display the frequencies of item thresholds (lower half) and respondents (upper half). High scores imply higher OD severity and low scores imply lower OD severity. **a** The J-EAT-10 responses of the full sample and **b** the J-EAT-10 responses grouped as dependent and independent respondents. For both graphs, the item thresholds spread over about 7 logits, with evidence of floor effects (a high percentage of respondents achieved the lowest possible score of zero), but not ceiling effects. Some item-thresholds are in the same place, which indicates that they are duplicating the ability to discriminate at that level of difficulty. Some areas along the logit scale are not represented by item thresholds

The J-EAT-10 scale presented poor targeting, with insufficient match between overall spread of items and spread of respondents, as illustrated in Fig. 3. There are many gaps on the item-thresholds continuum, indicating that the scale is not able to detect small changes in respondents across the whole continuum of OD severity. Some item-thresholds are in the same place. For example, around the location logit of − 1, the frequency of five thresholds is made up of item 3 (liquids effort), item 4 (solids effort), item 6 (painful), item 7 (pleasure eat), and item 10 (stressful). This indicates that these items are duplicating the ability to discriminate at that level of difficulty. Fig. 3a) shows that the 42% extreme scores relate to respondents giving a score of 0 (no problem) across all ten items of J-EAT-10 (i.e., floor effects). No respondents gave a score of 4 (severe problems) to all items (i.e., no ceiling effects). Figure 3b) displays the mean (SD) location for the dependent and independent respondents, which illustrates that the dependent respondents reported higher degrees of OD severity and are slightly more spread across the continuum, though still poorly aligned with the item spread.

### Improvement strategies

The improvement strategies were applied to the responses from the full sample. The fact that few items had ordered thresholds, argued for changing the response categories consistently for all items. Cordier et al. [16] suggests that the response scale should be changed from 5 to 3 points by combining scores 0 and 1 as well as scores 3 and 4, resulting in the scoring structure 00122. As seen in Table 1 (analysis 4), this produced more respondents at the extremes (65%), decreased the reliability and power of fit, and did not provide overall model or item level fit. Since the pattern of the category probability curves could argue for a three-score category solution, additional scoring structures were analyzed. None of these provided overall model fit (Table 1, analyses 5–8), and only two (analyses 5 and 6) did not produce more respondents at the extremes and maintained good power of fit. Further improvement strategies did not provide overall model fit for any of the proposed scoring structures. For illustrative purpose, Table 5 presents one of the attempts based on the scoring structure 01122. The summary fit residuals for items and persons improved during a stepwise removal of the most mis fitting items. However, the item-trait interaction remained significant, and the reliability and power of fit decreased markedly. Although the five retained items (items 1, 2, 5, 8 and 9) obtained acceptable fit residuals, the fit statistics remained significant.

**Table 5:** Fit statistics during removal of misfit items from J-EAT-10 with scoring structure 01122

Scale analysis	% Extr.	Fit residuals mean (SD)		Item-trait interaction		Reliability		Individual item-fit
		Item	Person	*χ*^2^ (df)	*P*	PSI / α	Power of fit	Misfit items
4.a. Remove item 6	42%	−1.58 (3.96)	−0.36 (0.95)	382.42 (36)	< 0.001	0.61 / 0.79	Reasonable	Item 1 / FR = 5.5^a,b^ Item 3 / FR = −4.3^a,b^ Item 4 / FR = −6.2^a,b^ Item 8 / FR = − 3.2^a,b^ Item 10 / FR = −6.4^a,b^ Item 2,5,7,9 ^a,b^
4.b. Remove item 10	42%	−1.28 (3.53)	− 0.34 (0.93)	299.55 (24)	< 0.001	0.53 / 0.74	Reasonable	Item 1 / FR = 4.6^a,b^ Item 3 / FR = − 4.5^a,b^ Item 4 / FR = − 6.2^a,b^ Item 8 / FR = − 3.1^a,b^ Item 2,5,7,9^a,b^
4.c. Remove item 4	43%	−1.02 (2.85)	−0.33 (0.87)	230.68 (21)	< 0.001	0.40 / 0.66	Low	Item 1/ FR = 3.5^a,b^ Item 3 / FR = − 4.9^a,b^ Item 8 / FR = − 2.6^a,b^ Item 2,5,7 ^a,b^
4.d. Remove item 3	43%	−0.69 (2.15)	− 0.32 (0.85)	196.29 (18)	< 0.001	0.24 / 0.55	Too low	Item 7 / FR = − 2.8 ^a,b^ Item 8 / FR = − 2.6 ^a,b^ Item 1,2,5 ^a,b^
4.e. Remove item 7	45%	−0.39 (1.63)	− 0.28 (0.75)	163.76 (10)	< 0.001	0.02 / 0.38	Too low	Item 1,2,5,8 ^a,b^
***Optimal values < 15%***		***0 (< 1.00)***	***0 (< 1.00)***		***> 0.05***	***> 0.70***		***FR < ±2.5, or non-significant fit statistics***

In analyses 4.c. to 4.e, item 9 displayed satisfactory fit

*J-EAT-10* Japanese version of the Eating Assessment Tool, *% Extr.* percentage of extreme scores, *SD* standard deviation, *χ*^*2*^ Chi-square, *df* degrees of freedom, *PSI* Person Separation Index, *α* Cronbach’s alpha, *CI* confidence interval

^a^Significant *χ*^2^ (Bonferroni adjusted)

^b^Significant F statistics (Bonferroni adjusted)

## Discussion

The current study presents a secondary analysis of existing data using the Rasch model. The aim was to evaluate whether measurements by J-EAT-10 are reliable, valid, and uphold specific objectivity when applied in OD screening in a non-clinical population of community-dwelling elders. Overall, the results align with the findings from clinical populations [16, 18] in terms of substantial floor effect and inappropriate targeting, disordered thresholds, several mis fitting items, unacceptable reliability by means of the PSI, but acceptable reliability by means of Cronbach’s α. However, the PSI should be used for interpretation of reliability, since these two reliability indices will diverge in the event of poor targeting and floor effect [27].

J-EAT-10 displayed inappropriate targeting and did not cover a high percentage of the sample, which on average presents a higher ability level than the average of the scale items. Although low physical performance and dependency are associated with OD [19, 21, 22], the inappropriate targeting was also present for the dependent respondents. The targeting problem and low PSI indicate that it is not possible to differentiate between different levels of OD when using J-EAT-10 as a screening tool in a non-clinical population in the wider community [27, 42]. In addition, the analyses revealed that the responses to most items are not consistent with the metric estimate of the latent variable, resulting in disordered thresholds. This suggest that the J-EAT-10 response structure does not function as intended when applied in a population-based survey. The improvement strategies for the response categories proposed by Cordier et al. [16] produced further extreme person scores, likely due to the frequent use of the score categories 0 and 1. Although fit to the Rasch model was not achieved, the best solutions appeared to be a three-point scoring structure with the pattern 01112 or 01122. This might indicate that meaningful differentiation of OD severity seems to be achievable with three response categories. It is worth noting that, besides too many response options, disordered thresholds might occur in the event of unclear or irrelevant item content and category descriptions or multidimensionality [27], which could occur in inadequately translated versions of PROMs [30]. In fact, Cordier et al. identified DIF by language for four translated versions of EAT-10 [16]. It cannot therefore be excluded that DIF by language exists for J-EAT-10. In the current study, there was no evidence of DIF by gender, age, or functional level. However, age is a continuous variable, stratified into four groups, and functional level was determined by care in minutes [22], which does not describe actual functional performance of older adults compared to information obtained by reliable and valid functional assessments [45]. Accordingly, further DIF analyses by language, age, and functional level might be needed.

The t-tests indicated unidimensionality, even though all the fit statistics indicated model misfit of J-EAT-10. Item 1 (lose weight) and item 9 (cough) displayed multidimensionality, and item 3 (liquids effort), item 4 (solids effort), item 6 (painful) and item 10 (stressful) showed high negative fit residuals, indicating redundancy, which was also reflected by the clustering of item-thresholds on the logit scale, as illustrated in Fig. 3. Though unidimensionality is a matter of degree and some level of item misfit might be unavoidable [27, 44], a proportion of 60% misfit items suggests that further examination of J-EAT-10 is needed. In order to find improvement strategies, misfit items were removed, resulting in a scale with item 1 (lose weight), item 2 (go out for meals), item 5 (pills effort), item 8 (stick throat), and item 9 (cough). Although the item fit-residuals improved, the item-trait interaction persisted to be significant, which indicates lack of homogeneity. In addition, the PSI became too low and four items persisted to display significant fit statistics. Accordingly, it cannot be recommended summarizing the item responses of J-EAT-10 into a total score when applied to a non-clinical population.

It is worth noting that misfit items should not be removed from a scale purely for statistical reasons without theoretical considerations, as this might distort the content validity of the measurement [27, 30]. Content validity is an important property of a PROM and refers to the degree to which the content of an instrument is relevant, comprehensive, and comprehensible with respect to the variable of interest and the target population [46]. The decision as to whether a scale is sufficiently unidimensional should ultimately therefore come from a synthesis of statistical analysis in conjunction with the purpose of measurement and clinical/theoretical considerations [27, 30]. Unfortunately, content validity is not established for either the original version of EAT-10 [47] nor J-EAT-10 [31], which restricted nuanced decisions for improvement strategies.

### Methodology considerations

Application of secondary analysis on an existing dataset, had an advantage and some disadvantages [48]. The advantage was that it was possible at relatively low cost to contribute to the knowledge base of the psychometric properties of J-EAT-10 using analysis by the Rasch model, which requires a relatively large dataset [43, 44]. The disadvantages were that the data were not collected for the purpose of analysis by the Rasch model and that not being involved in the data collection procedure might have meant that some study-specific aspects were concealed. Since the variables in the dataset were given, DIF analysis of important variables, such as disease state and a manifest diagnosis of OD, was not possible. In addition, the codebook for the dataset did not contain information on some of the tabulated values for the variable ‘functional level’. Accordingly, we decided not to include these in the analysis. Furthermore, the sample was skewed to low distributions of OD measured with J-EAT-10, resulting in a high percentage of extreme person scores and poor targeting, which influenced the effective sample size [27, 43]. Although an effective sample size of *N* = 661 (full sample) is regarded as sufficiently large [44], the data was still skewed toward the low distributions of OD. Considering an OD prevalence of 15% among community-dwelling elders [4], this might not be surprising. Accordingly, it could be argued in favor of not performing analysis by the Rasch model on item responses from non-clinical populations answering a PROM designed for a clinical population [30]. However, since EAT-10 is promoted as a quick and easy OD screening method [12] and routine screening of community-dwelling elders using EAT-10 is recommended [1–3, 10, 19–23], it was important to undertake current analysis by the Rasch model.

## Conclusion

The study adds knowledge to the evidence on the psychometric properties of a translated version of EAT-10. When J-EAT-10 was applied to detect OD in community-dwelling elders with low OD prevalence rates, it performed less than optimally. The main problems were substantial floor effect, low reliability, a rating scale not working as intended, and several redundant items. Different improvement strategies could not resolve the identified problems. Use of J-EAT-10 in population-based surveys cannot therefore be recommended. For such purpose, alternative screening tools of self-perceived OD should be chosen or a new one should be developed and validated.

## Supplementary information

**Additional file 1.** English version of EAT-10.

**Additional file 2.** Data codes of the existing data set and recodes for the analysis by the Rasch model.

## Data Availability

The dataset analyzed during the current study is available as supporting information (S1 File. data set) in Igarashi K, Kikutani T, Tamura F [10.1371/journal.pone.0211040.s001] [32].

## Abbreviations

ANOVA: Analysis of variance
CI: Confidence interval
df: degrees of freedom
DIF: Differential item functioning
EAT-10: Eating Assessment Tool
FR: Fit residual
J-EAT-10: Japanese version of EAT-10
LID: Local item dependence
Loc: Location
OD: Oropharyngeal dysphagia
PCM: Partial Credit Model
PSI: Person Separation Index
PROM: Patient reported outcome measure
RSM: Rating Scale Model
SD: Standard deviation
SE: Standard error
