# Supplementary material for: Item analysis of the Eating Assessment Tool (EAT-10) by the Rasch model: a secondary analysis of cross-sectional survey data obtained among community-dwelling elders
Source: Health Qual Life Outcomes. 2020 May 13;18:139. doi: 10.1186/s12955-020-01384-2 (PMC7222581; doi:10.1186/s12955-020-01384-2)
Supplement: Supplementary file 1 — Additional file 1. English version of EAT-10. [file 12955_2020_1384_MOESM1_ESM.docx]

Supplemental file: English version of EAT-10

*Note*. Accessed and retrieved 12-04-2019 from the Nestlé Nutrition Institute website (https://www.nestlenutrition-institute.org/resources/nutrition-tools/details/swallowing-assessment-tool.)
