# Supplementary material for: Item analysis of the Eating Assessment Tool (EAT-10) by the Rasch model: a secondary analysis of cross-sectional survey data obtained among community-dwelling elders
Source: Health Qual Life Outcomes. 2020 May 13;18:139. doi: 10.1186/s12955-020-01384-2 (PMC7222581; doi:10.1186/s12955-020-01384-2)
Supplement: Supplementary file 2 — Additional file 2. Data codes of the existing data set and recodes for the analysis by the Rasch model. [file 12955_2020_1384_MOESM2_ESM.docx]

Supplemental file 2: Data codes of the existing data set and recodes for the analysis by the Rasch model.

| Variables | Codes according to Igarashi et al (4) | Codes for the analysis by the Rasch model (sample size) |
| --- | --- | --- |
| ID number | 1-1878 | 1-1144 |
| Age in years | Continuous data from 60-99 | Stratified by quartiles for DIF analysis  1 = ≤ Q1 ~ 60-70 years (N = 313)  2 = ≤ Q2 ~ 71-77 years (N = 298)  3 = ≤ Q3 ~ 78-83 years (N = 263)  4 = > Q ~ 384-99 years (N = 270) |
| Gender | 1= male  2= female | Dichotomized for DIF analysis  1 = male (N = 475)  2 = female (N = 669) |
| Functional level | 11-16 = independent (stratified into six groups by age in years: 11 = (60-64), 12 = (65-69), 13 = (70-74), 14 = (75-79), 15 = (80-84), 16 = (85-89)).  17-23 = dependent (stratified into seven groups by care need in minutes per day: 17 = (25-31 minutes), 18 = (32-49 minutes / preventive support), 19 = (32-49 minutes), 20 = (50-69 minutes), 21 = (70-89 minutes), 22 = (90-109 minutes), and 23 = (≥110 minutes)).  24-27 = no codes provided. | Dichotomized for overall analysis by the Rasch model  1 = independent (codes 11-16) (N = 594)  2 = dependent (codes 17-23) (N = 550)  Stratified into four groups for DIF analysis  1 = independent (codes 11-16) (N = 594)  2 = low care (codes 17-21) (N = 265)  3 = moderate care (code 22) (N = 147)  4 = high care group (code 23) (N = 138).  Values 24-27 assigned to N = 353 were excluded as no codes were provided. |
| J-EAT-10 item 1 | 0 = No problem to 4 = Severe problem | 0 = No problem to 4 = Severe problem |
| J-EAT-10 item 2 | 0 = No problem to 4 = Severe problem | 0 = No problem to 4 = Severe problem |
| J-EAT-10 item 3 | 0 = No problem to 4 = Severe problem | 0 = No problem to 4 = Severe problem |
| J-EAT-10 item 4 | 0 = No problem to 4 = Severe problem | 0 = No problem to 4 = Severe problem |
| J-EAT-10 item 5 | 0 = No problem to 4 = Severe problem | 0 = No problem to 4 = Severe problem |
| J-EAT-10 item 6 | 0 = No problem to 4 = Severe problem | 0 = No problem to 4 = Severe problem |
| J-EAT-10 item 7 | 0 = No problem to 4 = Severe problem | 0 = No problem to 4 = Severe problem |
| J-EAT-10 item 8 | 0 = No problem to 4 = Severe problem | 0 = No problem to 4 = Severe problem |
| J-EAT-10 item 9 | 0 = No problem to 4 = Severe problem | 0 = No problem to 4 = Severe problem |
| J-EAT-10 item 10 | 0 = No problem to 4 = Severe problem | 0 = No problem to 4 = Severe problem |
| J-EAT-10 total score | 0 to 40 | Not included in analysis |
|  |  | J-EAT-10 with incomplete responses excluded from analysis (N = 378) |
